# Salvage lung resection after immunotherapy is feasible and safe

**DOI:** 10.1016/j.xjon.2024.03.018

**Published:** 2024-04-23

**Authors:** Attila Nemeth, Maureen E. Canavan, Peter L. Zhan, Brooks V. Udelsman, Sora Ely, Dennis A. Wigle, Linda Martin, Chi-Fu Jeffrey Yang, Daniel J. Boffa, Andrew P. Dhanasopon

**Affiliations:** a Department for Thoracic and Cardiovascular Surgery, University Hospital Tübingen, Tübingen, Germany; b Department of Internal Medicine, Cancer Outcomes Public Policy and Effectiveness Research Center, Yale University School of Medicine, New Haven, Conn; c Section of Thoracic Surgery, Department of Surgery, Yale University School of Medicine, New Haven, Conn; d Division of Thoracic Surgery, Department of Surgery, Mayo Clinic College of Medicine and Science, Rochester, Minn; e Division of Thoracic and Cardiovascular Surgery, Department of Surgery, University of Virginia Health System, Charlottesville, Va; f Division of Thoracic Surgery, Department of Surgery, Massachusetts General Hospital and Harvard Medical School, Boston, Mass

**Keywords:** lung cancer, immunotherapy, chemotherapy, radiation, lobectomy, pneumonectomy

## Abstract

**Objectives:**

Patients with non–small cell lung cancer treated with immunotherapy and modern chemoradiation regimens show improved progression-free and overall survival. However, patients with limited oligo-progression represent a potential population in which local therapy such as surgery may have a potential role as salvage treatment. The objectives of our study were to evaluate the feasibility and safety of salvage lung resection after immunotherapy in patients with non–small cell lung cancer.

**Methods:**

The National Cancer Database was queried for patients diagnosed and treated for non–small cell lung cancer stage I to IV, from 2013 to 2020. Patients who underwent surgery as salvage after immunotherapy were defined as undergoing surgery >5 months from the initiation of immunotherapy. As a sensitivity analysis, patients who underwent surgery as salvage after chemoradiation were also analyzed in a similar fashion. Surgical outcomes such as type of surgery, complete resection (R0) rates, and complete pathologic response rates were determined for feasibility. Length of stay, 30-day readmission rates, and 30-day mortality rates were determined and overall survivals were estimated with Kaplan-Meier analysis to evaluate for safety.

**Results:**

Of the 934,093 patients diagnosed with non–small cell lung cancer stage I to IV from 2013 to 2020, 164 patients received immunotherapy and after 5 months underwent surgery. Lobectomy was the most commonly performed operation (74%) and pneumonectomy was required in 9% (n = 15). R0 resection was achieved in 89% (n = 146) and of these patients, 23% (n = 37) had complete pathologic response. Median length of stay was 4 days, 30-day readmission was 5%, and 30-day mortality was 0.6%. In our sensitivity analysis of chemoradiation patients (n = 445), the above data were similar to previously reported cohort studies of patients undergoing chemoradiation and subsequently salvage surgery.

**Conclusions:**

Lung resection after immunotherapy appears to be a feasible salvage treatment option, with lobectomy being most common and with high R0 resection rates. Low patient morbidity and mortality rates also suggest the safety of this approach. Salvage surgery may be considered in patients who have oligo-progression after immunotherapy within the context of a comprehensive multidisciplinary treatment plan.

## Graphical Abstract

**Figure:**
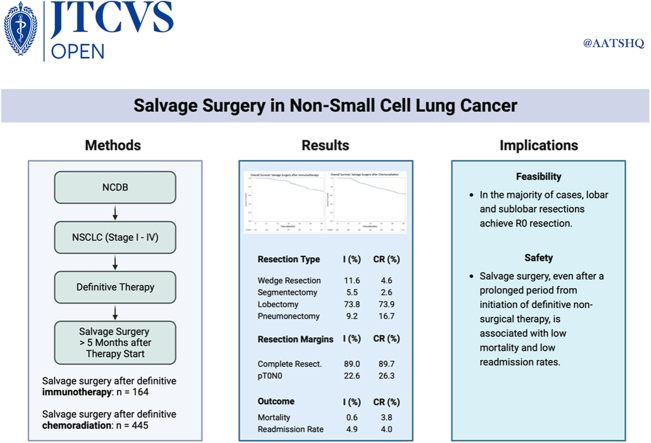

**Figure:**
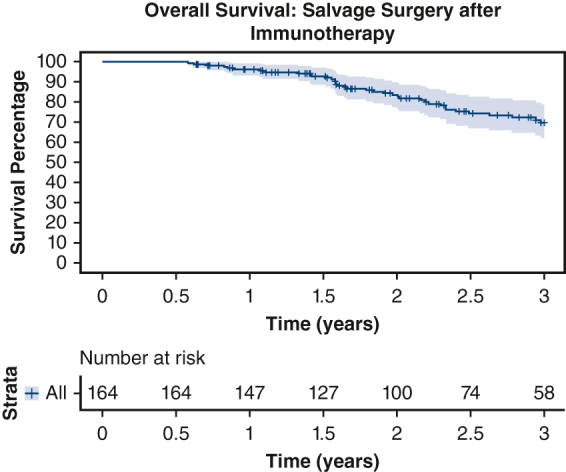
Overall survival: Salvage surgery after immunotherapy.

**Central Message**

Salvage lung resections are feasible and safe for patients with non–small cell lung cancer who have residual or recurrent tumors after immunotherapy and is associated with durable survival.

**Perspective**

Immunotherapy is being increasingly used to treat non–small cell lung cancer. However, more than half of patients who initially respond will progress within 7 months. For those patients with oligo-progression, surgery may be a viable option for eliminating residual or recurrent disease. Our NCDB study suggests that surgery can be a feasible and safe local therapy option in this setting.

See Discussion on page 151.

Immunotherapy has fundamentally transformed the management of locally advanced non–small cell lung cancer (NSCLC) with improved effectiveness of drug therapies, such as immune checkpoint inhibitors.^1^ The addition of immunotherapy to platinum-based chemotherapy provides an even superior pathological response and survival benefit over chemotherapy alone.^2^^,^^3^ Despite advances in multimodal therapies, many patients with locally advanced NSCLC experience local disease recurrence or local progression after definitive nonsurgical treatment.^4^^,^^5^ Cohort studies have suggested salvage surgery after chemoradiation after chemoradiation as a potential curative treatment option for these select patients, improving overall survival (OS) and quality of life. However, salvage surgery is also often associated with higher morbidity and mortality rates compared with primary surgery.^6^, ^7^, ^8^, ^9^

This study aimed to investigate the feasibility and safety of salvage lung resection in patients with NSCLC who received immunotherapy or definitive chemoradiation. The purpose of this study was to determine whether salvage lung resection could be a viable treatment option for this patient population given the potential benefits and risks associated with this procedure.

## Materials and Methods

**Figure 1:**
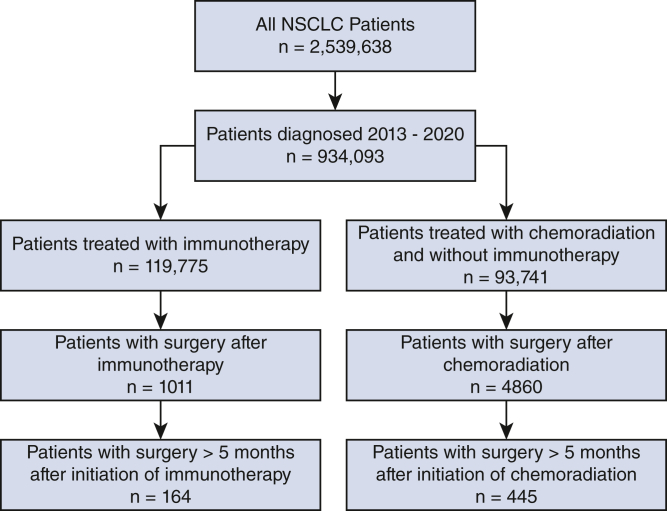
Patient selection. *NSCLC*, Non–small cell lung cancer.

The National Cancer Database (NCDB) was queried for adult patients diagnosed with NSCLC from 2013 to 2020, stages I through IV, treated with immunotherapy or chemoradiation, who subsequently underwent surgery (Figure 1). To exclude patients treated with neoadjuvant strategies, surgery as salvage was defined as surgery occurring at least 5 months after the initial immunotherapy treatment or initial chemoradiation treatment.^10^^,^^11^

### Data Source and Study Design

Data were obtained from the NCDB, a large-scale registry that captures information on cancer cases from more than 1500 Commission on Cancer Centers in the United States and Puerto Rico. The NCDB is maintained in collaboration with the American Cancer Society and the American College of Surgeons and is estimated to capture approximately 70% of newly diagnosed cases of cancer in the United States. Certified independent tumor registrars used standardized coding guidelines to ensure the quality and accuracy of the data captured in the NCDB. The NCDB provides extensive clinical and demographic information on patients treated at Commission on Cancer-approved hospitals, including diagnosis, stage of diagnosis, primary treatment, follow-up information, and other relevant data. It captures 72% of all newly diagnosed malignancies in the United States annually. Overall coverage of cancer cases in the NCDB has remained relatively stable with a slight increase from 67% observed during the period from 2004 to 2006. Case coverage also increased slightly between 2012 and 2014, as did the number of Commission on Cancer-accredited facilities, which increased from 1455 to 1475 and represents approximately 25% of acute-care facilities. Data captured in the NCDB were recorded using the American Joint Committee on Cancer's seventh edition TNM classification.

It is important to note that the analytical or statistical methods used as well as the conclusions drawn from the data obtained from the NCDB have not been verified by the American College of Surgeons or the Commission on Cancer; therefore, they are not accountable for them.

This study, utilizing de-identified data from the NCDB, was reviewed by the Institutional Review Board of Yale University and was exempt from informed consent requirements because the data were fully de-identified.

### Patient Selection

This study aimed to evaluate the clinical outcomes of adult patients diagnosed with NSCLC in the NCDB between 2013 and 2020. Our study was a retrospective analysis of data obtained from the NCDB, focusing on the demographic, clinical, and pathological characteristics of the study cohort.

The inclusion criteria for the study were adults aged 18 years or older with a diagnosis of NSCLC, whereas the exclusion criteria were patients who underwent primary surgery, those who never underwent surgery, or if survival data were unknown. Additional exclusions were made for cases in which information on the surgical procedure was missing or in which ablative procedures were performed.

Variables such as age, sex, ethnicity, comorbidities (represented by the Charlson/Deyo score), primary tumor site, clinical stage, histology, grade, definitive therapy, and procedure type were extracted from the NCDB. The study used the seventh edition of the American Joint Committee on Cancer TNM staging criteria to report the clinical and pathological stages. Histology was simplified into 2 categories, adenocarcinoma and nonadenocarcinoma, as classified by the International Classification of Disease for Oncology, third edition.

The treatment sequence of surgery and chemotherapy was defined using the NCDB variables, with the extent of surgery documented as sublobar resection (wedge resection or segmentectomy), lobectomy, or pneumonectomy. We defined definitive surgery as noted by the NCDB patient user file data dictionary to be the most invasive surgical procedure for the primary site. Immunotherapy was defined as first course treatment using biological or chemical agents that alter the immune system or change the host's response to tumor cells. Therapy was grouped into immunotherapy with or without chemoradiation, and chemoradiation without immunotherapy. Definitive chemoradiation was characterized by the combination of multiagent chemotherapy and radiation therapy. Our study design intentionally did not aim for a direct comparison between the immunotherapy and chemoradiation groups, recognizing the absence of a traditional control group. This approach was chosen to focus on evaluating the outcomes and feasibility within each treatment modality independently. Complete pathologic response was defined as pathologic stage pT0 N0. All other categories, including no pathologic response or partial pathologic response, were classified as not pT0 N0.

To address the potential for neoadjuvant therapy bias, we utilized a conditional landmark analysis, setting the surgery time point to at least 5 months after the initiation of immunotherapy, chemotherapy, and radiotherapy. The primary outcome of the study was 3-year overall survival, with secondary outcomes, including 30-day and 90-day mortality, hospital length of stay, 30-day readmission rate, R0 resection, and extent of surgery. These secondary outcomes were examined as potential surrogate markers for postoperative morbidity and feasibility of surgical therapy, which were not recorded in the NCDB.

### Statistical Analysis

Before the analysis, the normality of the data was evaluated using the Shapiro-Wilk test. Continuous variables were expressed as median (interquartile range [IQR]), whereas categorical variables were presented as absolute numbers and percentages. To compare continuous variables between groups, Mann-Whitney *U* test was utilized, whereas categorical variables were assessed using χ^2^ test. For the construction of survival curves, the Kaplan-Meier method was employed, and their comparability was assessed using the log-rank test. To model the relationship between surgery type and timing and mortality, logistic regression analysis was executed. The statistical models used in this study were not adjusted for the covariates. All statistical analyses were performed using SAS (version 9.4) manufactured by Statistical Analysis System Institute.

## Results

A total of 609 patients with stage I through IV NSCLC who underwent salvage surgery after immunotherapy (n = 164) or definitive chemoradiation therapy (n = 445) were identified in the NCDB from 2013 to 2020. Patient characteristics are presented in Table 1. There was no statistically significant difference in the sex distribution between the 2 groups. Specifically, 45.1% of patients in the immunotherapy group were men compared to 54.9% women, whereas 53.4% of patients in the chemoradiation group were men compared with 46.6% women. The median age was 61 years (IQR, 56-68 years) in the immunotherapy group and 62 years (IQR, 55-69 years) in the chemoradiation group. Most patients in the immunotherapy group had stage III (39.6%) and IV (34.2%) disease, whereas most patients in the chemoradiation group had stage II (22.7%) and III (68.1%) disease. Data were missing for 6.1% of the patients in the immunotherapy group and 1.1% of the patients in the chemoradiation group. Most patients in the immunotherapy group had adenocarcinoma (70.1%), whereas in the chemoradiation group, there was an even distribution of adenocarcinoma (47.0%) and nonadenocarcinoma (53.0%). Comorbidity, measured using the Charlson comorbidity score, was similar between the 2 groups. In the immunotherapy group, 67.7% of patients had a score of 1 and 23.2% had a score of 2. In the chemoradiation group, 58.4% of patients had a score of 1 and 27.4% had a score of 2. See Table 1.

**Table 1:** Baseline characteristics and postsurgical outcomes among patients who underwent salvage surgery after immunotherapy or chemoradiation

Characteristic	Immunotherapy	Chemoradiation
	(n = 164)	(n = 445)
Age (y)	61 (56-68)	62 (55-69)
Sex		
Male	74 (45.1)	243 (53.4)
Female	90 (54.9)	212 (46.6)
Race		
Black	15 (9.2)	46 (10.1)
White	134 (87.7)	384 (84.4)
Other	15 (9.2)	25 (5.5)
Histology		
Adenocarcinoma	115 (70.1)	214 (47.0)
Squamous	38 (23.2)	199 (44.7)
Large cell	0 (0.0)	9 (2.0)
Other	7 (4.3)	23 (5.2)
BAC	4 (2.4)	0 (0.0)
Clinical stage		
1	16 (9.8)	20 (5.5)
2	17 (10.4)	101 (22.7)
3	65 (39.6)	303 (68.1)
4	56 (34.2)	16 (3.6)
Missing	10 (6.1)	5 (1.1)
Surgical procedure		
Wedge resection	19 (11.6)	21 (4.6)
Segmentectomy	9 (5.5)	12 (2.6)
Lobectomy	121 (73.8)	336 (73.9)
Pneumonectomy	15 (9.2)	76 (16.7)
R0 Resection	146 (89.0)	399 (89.7)
Complete pathologic response: pT0 N0	37 (22.6)	117 (26.3)
Not complete pathologic response	146 (89.0)	399 (89.7)
Treatment		
Chemotherapy	148 (90.3)	
Radiation	63 (38.4)	
Time of surgery after immunotherapy (mo)		
5-6	51 (31.1)	211 (46.4)
6-7	41 (25.0)	121 (26.6)
7-9	42 (25.6)	86 (18.9)
>9	30 (18.3)	37 (8.1)
Charlson comorbidity score		
1	111 (67.7)	260 (58.4)
2	38 (23.2)	122 (27.4)
3	11 (6.7)	40 (9.0)
4	4 (2.4)	23 (5.2)
30-d Mortality	1 (0.6)	17 (3.8)
90-d Mortality	7 (4.3)	35 (7.9)
Readmission within 30 d	8 (4.9)	18 (4.0)
Length of stay (d)	4.0 (2.0-6.0)	5.0 (3.0-7.0)

Values are presented as median (interquartile range) or overall (%). *BAC*, Bronchioloalveolar carcinoma.

**Figure 2:**
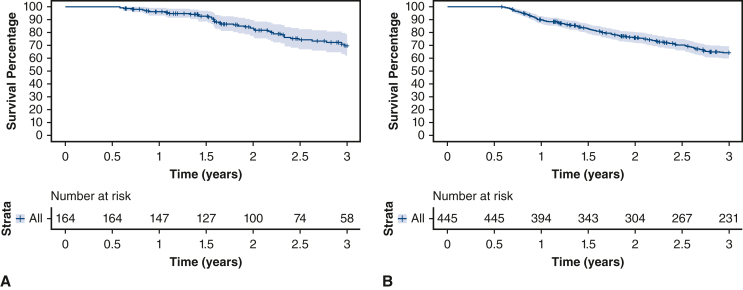
A, Overall survival of patients with non–small cell lung cancer (*NSCLC*) (stages I-IV) who underwent salvage surgery after immunotherapy. Three-year overall survival: 73.4%. Stratum-specific 95% CIs are presented by the shaded region. B, Overall survival of patients with NSCLC (stages I-IV) who underwent salvage surgery after chemoradiation. Three-year overall survival: 69.8%. Stratum-specific 95% CIs are presented by the shaded region.

**Figure E1:**
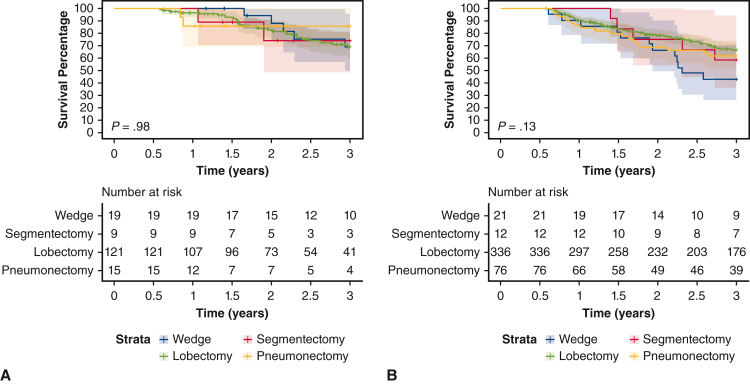
A, Overall survival of patients with non–small cell lung cancer (*NSCLC*) (stages I-IV) who underwent salvage surgery with different extents of resection after immunotherapy. Three-year overall survival: lobectomy (72.5%), pneumonectomy (92.9%), segmentectomy (74.1%), wedge (69.0%). Stratum-specific 95% CIs are presented by the shaded region. B, Overall survival of patients with NSCLC (stages I-IV) who underwent salvage surgery with different extents of resection after immunotherapy. Three-year overall survival: lobectomy (72.3%), pneumonectomy (64.7%), segmentectomy (58.3%), wedge (42.9%). stratum-specific 95% CIs are presented by the shaded region.

Additionally, we evaluated the 30-day mortality rate, unplanned readmissions to the hospital, and length of hospital stay in both groups. In the immunotherapy group, the 30-day mortality rate was 0.6%, and the rate of unplanned hospital readmissions within 30 days was 4.9%. The median length of stay was 4 days (IQR, 2.0-6.0 days). In contrast, the 30-day mortality rate in the chemoradiation group was 3.8%, and the rate for unplanned hospital readmissions within the same period was 4.0%. The median length of stay was 5 days (IQR, 3.0-7.0 days). The OS of both groups was compared using Kaplan-Meier curves, and the results are presented in Figure 2. Figure 2, *A* and *B*, shows the OS of the immunotherapy and chemoradiation groups, respectively (see Figure E1 for the OS of different extents of resection after immunotherapy).

Among the patients in the immunotherapy group, the majority (31.1%) underwent surgery 5 to 6 months after the beginning of immunotherapy. One-quarter of the patients (25.0%) underwent surgery 6 to 7 months after the start of immunotherapy, 25.6% underwent surgery in the 7- to 8-month range, and 18.3% underwent surgery more than 9 months later. Most patients (46.4%) underwent surgery 5 to 6 months after the start of chemoradiation treatment. Of the remaining patients, 26.6% underwent surgery after 6 to 7 months, 18.9% underwent surgery in the 7- to 9-month range, and 8.1% underwent surgery more than 9 months after starting treatment.

We performed logistic regression analysis to investigate the relationship between the timing of surgery after the initiation of therapy and 90-day mortality in our study population. The reference group comprised patients who underwent surgery between 5 and 6 months after the start of chemoradiation treatment. Logistic regression analysis showed that patients who underwent surgery 6 to 7 months after therapy initiation had an odds ratio (OR) of 1.650 (*P* = .823) for 90-day mortality. Similarly, those who underwent surgery 7 to 8 months later had an R of 1.603 (*P* = .916), whereas those who underwent surgery 9 months or later had the highest OR 2.192 (*P* = .369) for 90-day mortality. In a separate analysis, we investigated the relationship between the timing of surgery and the 90-day mortality in the immunotherapy group. Logistic regression analysis revealed that patients who underwent surgery 6 to 7 months after initiating immunotherapy had an OR of 0.621 (*P* = .917) for 90-day mortality, whereas those who underwent surgery between 7 and 9 months had an OR of 0.742 (*P* = .763). Additionally, patients who underwent surgery 9 months or later after the initiation of immunotherapy had the lowest OR for 90-day mortality, with a value of 0.391 (*P* = .383). See Table 2.

**Table 2:** Logistic regression model of the timing of surgery after immunotherapy and chemoradiation with the 90-day mortality outcome

Predictor	Odds ratio	95% CI		*P* value
		Lower	Upper	
Timing of surgery after immunotherapy (mo)∗				
>6-7	0.621	0.175	2.204	.917
>7-9	0.742	0.223	2.472	.763
>9	0.391	0.077	1.988	.383
Timing of surgery after chemoradiation (mo)∗				
>6-7	1.650	0.758	3.590	.823
>7-9	1.603	0.671	3.830	.916
>9	2.192	0.744	6.460	.369

∗ Reference = 5 to 6 months.

### Type of Surgery

The most common surgical procedure in the immunotherapy group was lobectomy (73.8%), followed by wedge resection (11.6%), pneumonectomy (9.2%) and segmentectomy (5.5%). Similarly, in the chemoradiation group, lobectomy was performed in 73.9% of the patients, followed by pneumonectomy (16.2%), wedge resection (4.6%), and segmentectomy (2.6%). Regarding surgical margins, 89.0% of patients in the immunotherapy group and 89.7% of patients in the chemoradiation group underwent R0 resection. Subgroup analysis of both groups showed that 22.6% of R0-resected patients in the immunotherapy group had pathological T0 stage, whereas in the chemoradiation group, 26.3% had pathological T0 stage.

### Chemotherapy and Radiation

In the immunotherapy cohort, 84% of the patients underwent multiagent chemotherapy, with 39% receiving radiotherapy, primarily at the tumor site (71%). Conversely, all patients in the chemoradiation group received both treatments, with the vast majority (98.9%) receiving radiation at the primary site. The remaining 1.1% of patients received radiation directed at other sites, including the spine, vertebral bodies, soft tissue, or brain. See Table 3.

**Table 3:** Logistic regression model of type of surgery after immunotherapy and chemoradiation with the outcome 90-day mortality

Predictor	Odds ratio	95% CI		*P* value
		Lower	Upper	
Type of surgery after immunotherapy∗				
Wedge	<0.001	<0.001	>999.999	.947
Segmentectomy	0.82	0.09	7.39	.953
Pneumonectomy	1.77	0.45	6.92	.939
Type of surgery after chemoradiation∗				
Wedge	1.09	0.24	4.96	.964
Segmentectomy	<0.001	<0.001	>999.999	.962
Pneumonectomy	1.76	0.84	371	.957

∗ Reference = lobectomy.

Our patient population was subjected to logistic regression analysis to investigate the impact of the type of surgery and extent of resection on 90-day mortality. Lobectomy was the preferred surgical technique and was used as the reference group. In the immunotherapy group, we discovered that patients who underwent wedge resection had an OR < .001 (*P* = .947) for 90-day mortality, whereas those who underwent segmentectomy had an OR of .82 (*P* = .953). Pneumonectomy had an OR of 1.77 (*P* = .939) for 90-day mortality compared with the reference group. The lobectomy group was used as the reference group. Patients who underwent wedge resection had an OR of 1.09 (*P* = .964) for the 90-day mortality. In contrast, the segmentectomy group had a negligible OR < .001 (*P* = .962), indicating a significantly lower risk of 90-day mortality than the reference group. Pneumonectomy group patients had an OR of 1.76 (*P* = .957) for 90-day mortality.

## Discussion

Salvage surgery emerges as a promising treatment modality for NSCLC patients after unsuccessful definitive nonsurgical treatments, aiming not only at local tumor control but also at improving survival rates. Yet, its broader application remains tempered by concerns over morbidity and the nuanced efficacy observed across patient subsets.^12^ Does the survival advantage suggested by existing literature for salvage surgery, amidst its complexities, hold a consistent promise across diverse NSCLC scenarios? This study seeks to examine the feasibility and results of salvage surgery in the area where definitive treatments have failed.

Available studies suggest that salvage surgery may confer some survival advantage for selected NSCLC patients after various modalities of chemoradiation, tyrosine kinase inhibitors, and immune checkpoint inhibitors who have a good performance status, localized disease, favorable histology, complete resection, and no major complications.^6^^,^^13^, ^14^, ^15^, ^16^ However, these studies were primarily retrospective and observational, with small sample sizes and potential bias. Consequently, the efficacy and safety of salvage surgery in patients with NSCLC remain unclear, and more extensive, well-designed prospective studies are necessary to establish its usefulness. The selection criteria for patients eligible for salvage surgery after immunotherapy or chemoradiation are not well defined. Studies suggest that salvage surgery may be feasible and beneficial in selected patients who achieve a good response or stable disease after systemic or local treatment, and who have resectable tumors with no distant metastasis.^17^, ^18^, ^19^, ^20^

Salvage lung resection can be performed by either lobectomy or sublobar resection, with the extent of surgery being a crucial factor in determining the feasibility of salvage surgery. It is widely believed that most patients with locally advanced NSCLC require lobectomy or pneumonectomy to achieve adequate resection margins.^21^ Our study found that the most common procedure in both the immunotherapy and chemoradiation groups was lobectomy, accounting for 73.8% and 73.9% of the cases, respectively. Sublobar resections were performed in 17.1% of patients in the immunotherapy group, whereas pneumonectomy was performed in 16.7% of patients in the chemoradiation group. Complete resection was achieved in 89.0% of patients in the immunotherapy group and 89.7% of patients in the chemoradiation group. A review of salvage surgery resection after chemoradiation by Hamada and colleagues^21^ showed a mean pneumonectomy rate of 28%, with lobectomy performed in 63% of the cases. Even higher rates of lobectomy were observed in smaller studies of salvage surgery after immunotherapy or targeted therapy (78%-100%).^19^^,^^22^^,^^23^ Given the perioperative morbidity and mortality associated with pneumonectomies, it appears that performing a lobectomy or sublobar resection and achieving adequate negative margins is a feasible surgical management approach. To compare the procedures in terms of OS, we performed logistic regression with lobectomy as the reference in both groups. In the chemoradiation group, the OR for pneumonectomy was 1.76 (*P* = .957). In the immunotherapy group, the OR for pneumonectomy was 1.77 (*P* = .939). Although our study does not establish a significant survival difference between surgical procedures, it prompts a reevaluation of surgical strategies in NSCLC treatment, encouraging further research into how parenchymal-sparing procedures might influence long-term outcomes. Nonetheless, the OR suggests that patients in whom lobectomy and sublobar resection can be performed may have a potential advantage regarding OS over patients undergoing pneumonectomy. The observed trends, despite statistical caution due to a small patient cohort and heterogeneity, underline the necessity for larger, more controlled studies to explore the potential benefits of different surgical approaches. Our data indicate that lobectomy is not inferior to pneumonectomy in terms of survival outcome. The lower perioperative morbidity and mortality associated with lobectomy not only reinforce its feasibility but also suggest a paradigm shift in selecting surgical options, highlighting the importance of personalized patient care in NSCLC treatment.

In this study, a high rate of complete resection (R0) in both immunotherapy and chemoradiation groups were seen (89.0% and 89.7%, respectively). These findings align with those reported in the literature, where complete resection rates span from 81% to 100%, irrespective of the initial treatment approach.^7^^,^^8^^,^^16^^,^^21^, ^22^, ^23^ Notably, a significant segment of our cohort achieving complete resection also presented with pathologic T0 (pT0) stage—22.6% in the immunotherapy group and 26.3% in the chemoradiation group. In contrast, previous studies reported much lower pT0 stages, ranging from 6% to 27%, or did not report it at all.^7^^,^^16^^,^^22^

**Figure 3:**
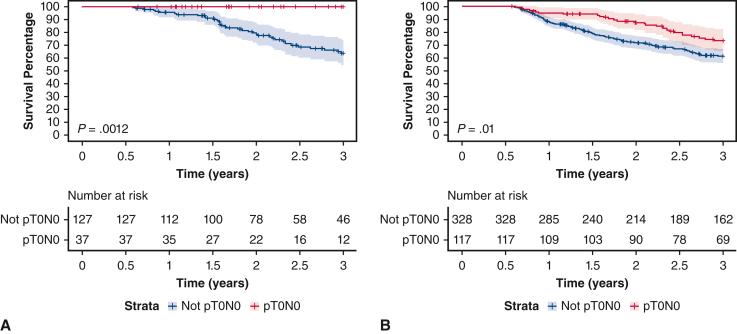
A, Overall survival of patients with non–small cell lung cancer (*NSCLC*) (stages I-IV) who underwent salvage surgery and R0 resection after immunotherapy. Three-year overall survival: pT0 N0 (100%), not pT0 N0 (67.2%). Stratum specific 95% CIs are presented by the shaded region. B, Overall survival of patients with NSCLC (stages I-IV) who underwent salvage surgery and R0 resection after chemoradiation. Three-year overall survival: pT0 N0 (77.5%), not pT0 N0 (66.7%). Stratum-specific 95% CIs are presented by the shaded region.

Figure 3 showcases a pivotal comparison of OS among patients who underwent complete resection with or without pT0. Patients who underwent immunotherapy prior to salvage surgery and reached pT0 stage showcased an OS rate of 100%, significantly surpassing the survival rate of 67.2% observed in patients with viable tumor resections. Similarly, in the chemoradiation group, patients with pT0 had a significantly better survival rate than those with resected viable tumors.

To assess the safety of salvage surgery, we analyzed the length of hospital stay, readmission rate, and mortality within the first 30 days after discharge. Patients who received immunotherapy had an average hospital stay of 4.0 days (IQR, 2.0-6.0 days), contrasting with 5.0 days (IQR, 3.0-7.0 days) for those undergoing chemoradiation. Early readmission rates were comparably low, at 4.9% in the immunotherapy group and 4.0% in the chemoradiation group. Postsurgery mortality rates were minimal, at 0.6% and 3.8% in the immunotherapy and chemoradiation groups, respectively, underscoring the procedure's safety within the initial postoperative period.

The length of hospital stay reported in our study, which varied from 4 to 9 days, is within the range reported in the literature.^8^^,^^16^^,^^19^^,^^22^ The 30-day mortality rate in the literature also varies widely, ranging from 0% to 11% and putting our study in the lower end of it.^7^^,^^8^ Salvage surgery is commonly associated with an increased incidence of perioperative complications. This is due to data from neoadjuvant chemoradiation therapies, which demonstrate fibrotic changes in hilar structures. Moreover, these patients usually have a greater tumor burden or are initially deemed inoperable.^24^, ^25^, ^26^ Another safety factor for patients is the extent of surgery, where an increased incidence of complications is associated with a higher rate of pneumonectomies.^27^ Our data regarding length of hospital stay, readmission rate, mortality, and the higher rate of lobar and sublobar resection indicate that salvage surgery may be a safer option than previously believed.

The optimal timing of salvage surgery remains uncertain. Our findings highlight a pivotal insight into the timing of salvage surgery following initial therapy for NSCLC, suggesting a nuanced interplay between early and late surgical interventions. Specifically, the data reveal that late salvage surgery after chemoradiation may be influenced by hilar fibrosis, whereas surgeries performed late after immunotherapy may benefit from its immunoalteration effects. This distinction underscores the need for future research to delve deeper into the biological influence of timing on salvage surgery outcomes, potentially guiding more personalized and effective treatment strategies for NSCLC patients. We categorized patients into 4 groups based on when they underwent salvage surgery following their initial therapy. This division enabled a closer look at the effects of timing on surgical outcomes after immunotherapy. Our goal was to enhance the study's consistency and minimize bias through this approach. Recognizing how these timing nuances influence outcomes is crucial for refining surgical schedules to improve patient results. Most of the patients in both the immunotherapy and chemoradiation groups underwent salvage surgery 5 to 7 months after the start of initial therapy. We chose 5 months as the cutoff point for salvage surgery due to the lack of data on the time frame between the start of initial therapy and surgery and to exclude patients who did not meet our criteria. Our data showed that a longer time frame between the initial therapy and salvage surgery was prognostically better in patients who received immunotherapy, with an OR of 0.391 for surgery >9 months after the start of immunotherapy. However, for patients who received chemoradiation, the opposite was observed, with an OR of 2.192 for surgery >9 months after the start of chemoradiation, although the results were not significant. Although longer intervals may increase the intraoperative risk, they can also confirm the absence of distant recurrences.^23^ Another important consideration is the possibility of developing drug resistance during the extended interval, which could influence the effectiveness of salvage surgery.^7^^,^^17^

### Limitations

The findings of this study should be interpreted with caution due to several important limitations. First, the sample size was insufficient for subgroup analysis, particularly in the immunotherapy group, although larger than other published studies. Second, the patients had diverse clinical stages and tumor histology, which limited the ability to compare therapy strategies in detail. Third, we lack data on the exact timing of initial treatment and salvage surgery. We used 5 months or later as a time point for salvage surgery, which could have resulted in selection bias and misclassification. For example, some patients in our cohort might have received neoadjuvant treatment, or there may be cases of patients who underwent salvage surgery earlier than 5 months that were not captured in our dataset. Fourth, we were unable to determine the specific immunotherapy regime; the dataset we used from the NCDB includes a range of agents under the immunotherapy variable, such as immune checkpoint inhibitors, tumor vaccines, and other immunomodulatory drugs. Therefore, drugs such as tyrosine kinase inhibitors could have been classified as immunotherapy in the NCDB dataset. Finally, our study lacked specific details regarding the indication for salvage surgery, including if it was for persistent disease, recurrence, or whether or not wedge resections were performed for diagnostic purposes. Furthermore, our dataset revealed the inclusion of early stage lung cancer patients undergoing immunotherapy. This subset predominantly comprised individuals initially deemed inoperable or those participating in clinical trials. Due to the limitations of our data source, specific details regarding the rationale for choosing immunotherapy in these early stage patients were not available. This aspect represents a notable limitation in our analysis because it precludes a comprehensive understanding of the decision-making processes underlying the treatment choices for these patients.

## Conclusions

**Figure 4:**
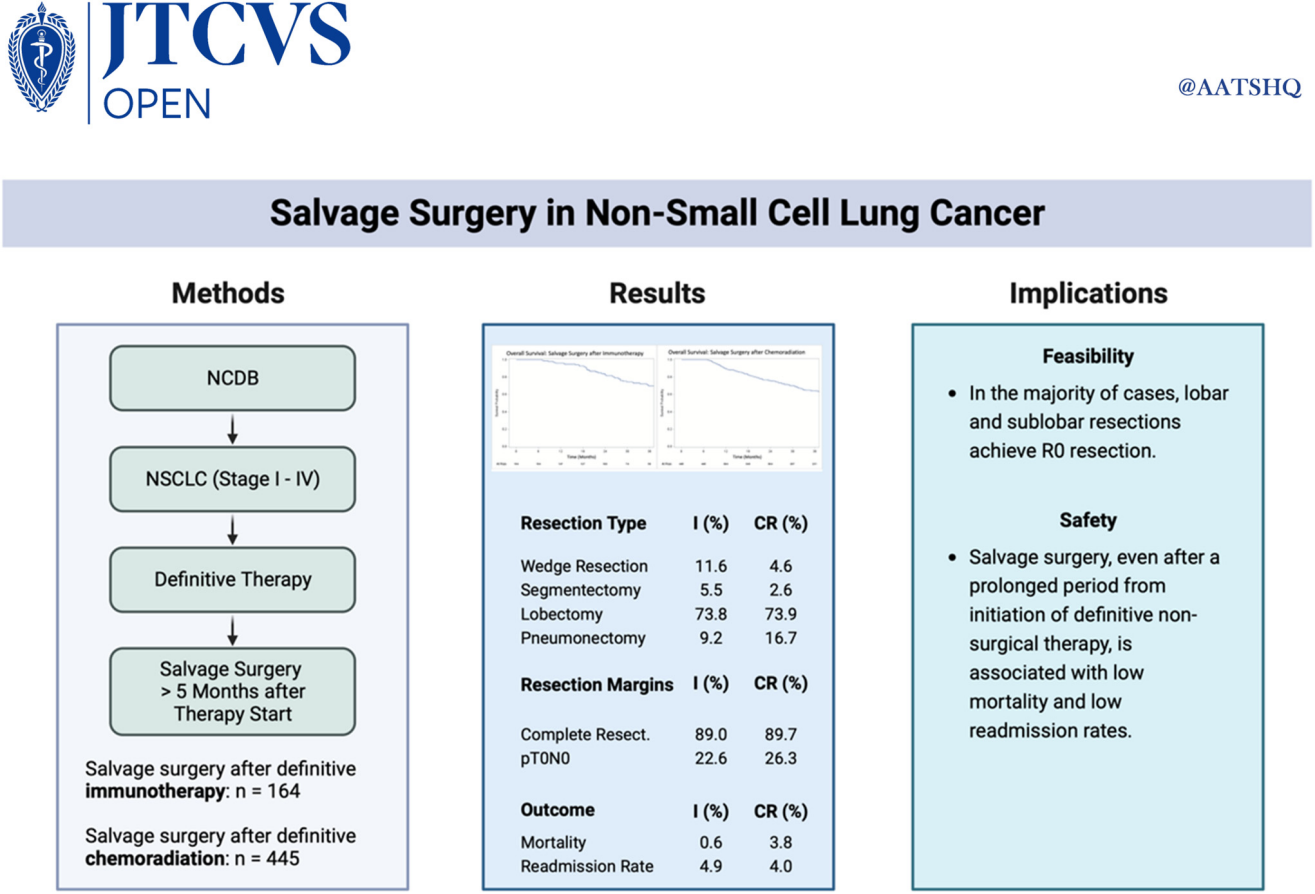
Graphical abstract. *NCDB*, National Cancer Database; *NSCLC*, non–small cell lung cancer; *I*, immunotherapy group; *CR*, chemoradiation group.

In this study, we analyzed the outcomes of salvage surgery in patients who had previously received definitive therapy strategies, such as immunotherapy or chemoradiation, for lung cancer using data from the NCDB (Figure 4). We found that salvage surgery was feasible and safe, with high rates of complete resection, short hospital stays, and low 30-day mortality and unplanned readmission rates. These results are encouraging and suggest that salvage surgery may offer a potential benefit for highly selected patients who have residual or recurrent disease after definitive therapy. Prospective randomized trials are needed to confirm the efficacy and optimal timing of salvage surgery in this patient population.

### Webcast

You can watch a Webcast of this AATS meeting presentation by going to: https://www.aats.org/resources/salvage-lung-resection-after-immunotherapy-in-lung-cancer-is-feasible-and-safe.

**Figure:**
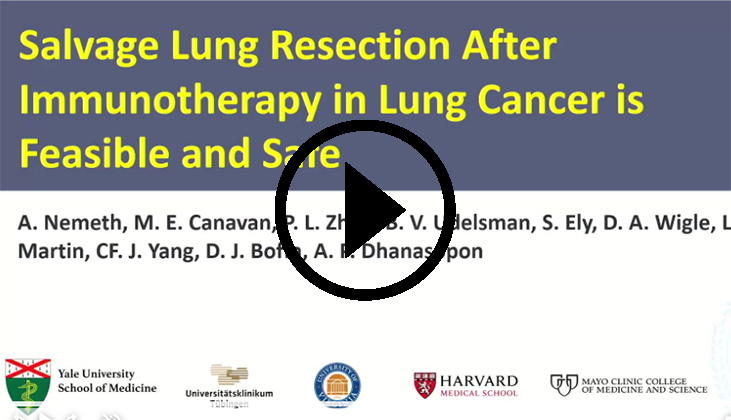

## Conflict of Interest Statement

The authors reported no conflicts of interest.

The *Journal* policy requires editors and reviewers to disclose conflicts of interest and to decline handling manuscripts for which they may have a conflict of interest. The editors and reviewers of this article have no conflicts of interest.

## References

[1] Ruiz-Cordero R., Devine W.P. (2020). Targeted therapy and checkpoint immunotherapy in lung cancer. Surg Pathology Clin.

[2] Heraudet L., Delon T., Veillon R. (2022). Effect of prior immunotherapy on the efficacy of chemotherapy in advanced non-small cell lung cancer: a retrospective study. Thorac Cancer.

[3] Reck M., Remon J., Hellmann M.D. (2022). First-line immunotherapy for non–small-cell lung cancer. J Clin Oncol.

[4] Friedes C., Mai N., Fu W. (2020). Propensity score adjusted analysis of patients with isolated locoregional recurrence versus de novo locally advanced NSCLC treated with definitive therapy. Lung Cancer.

[5] Kang J., Zhang C., Zhong W. (2021). Neoadjuvant immunotherapy for non–small cell lung cancer: state of the art. Cancer Commun.

[6] Sonobe M., Yutaka Y., Nakajima D. (2019). Salvage surgery after chemotherapy or chemoradiotherapy for initially unresectable lung carcinoma. Ann Thorac Surg.

[7] Shimada Y., Suzuki K., Okada M. (2016). Feasibility and efficacy of salvage lung resection after definitive chemoradiation therapy for Stage III non-small-cell lung cancer. Interact Cardiovasc Thorac Surg.

[8] Casiraghi M., Maisonneuve P., Piperno G. (2017). Salvage surgery after definitive chemoradiotherapy for non–small cell lung cancer. Semin Thorac Cardiovasc Surg.

[9] Ye J.C., Ding L., Atay S.M. (2020). Trimodality vs chemoradiation and salvage resection in cN2 stage IIIA non–small cell lung cancer. Semin Thorac Cardiovasc Surg.

[10] Provencio M., Nadal E., Insa A. (2020). Neoadjuvant chemotherapy and nivolumab in resectable non–small-cell lung cancer (NADIM): an open-label, multicentre, single-arm, Phase 2 trial. Lancet Oncol.

[11] Forde P.M., Spicer J., Lu S. (2022). Neoadjuvant nivolumab plus chemotherapy in resectable lung cancer. New Engl J Med.

[12] Uramoto H. (2016). Current topics on salvage thoracic surgery in patients with primary lung cancer. Ann Thorac Cardiovasc.

[13] Schreiner W., Dudek W., Lettmaier S., Fietkau R., Sirbu H. (2016). Should salvage surgery be considered for local recurrence after definitive chemoradiation in locally advanced non–small cell lung cancer?. J Cardiothorac Surg.

[14] Kaba E., Ozyurtkan M.O., Ayalp K., Cosgun T., Alomari M.R., Toker A. (2018). Salvage thoracic surgery in patients with lung cancer: potential indications and benefits. J Cardiothorac Surg.

[15] Bograd A.J., Mann C., Gorden J.A. (2020). Salvage lung resections after definitive chemoradiotherapy: a safe and effective oncologic option. Ann Thorac Surg.

[16] Suzuki S., Asakura K., Okui M. (Published online January 9, 2022). Safety and efficacy of salvage surgery for non–small cell lung cancer: a retrospective study of 46 patients from four Keio-affiliated hospitals. Gen Thorac Cardiovasc Surg.

[17] Shimizu K., Ohtaki Y., Suzuki K. (2021). Salvage surgery for non–small cell lung cancer after definitive radiotherapy. Ann Thorac Surg.

[18] Ohtaki Y., Shimizu K., Saitoh J.I. (2019). Is salvage surgery for patients with lung cancer after carbon ion radiotherapy easy or difficult?. Interact Cardiovasc Thorac Surg.

[19] Ohtaki Y., Shimizu K., Suzuki H. (2021). Salvage surgery for non–small cell lung cancer after tyrosine kinase inhibitor treatment. Lung Cancer.

[20] Suzuki S., Goto T. (2020). Role of surgical intervention in unresectable non-small cell lung cancer. J Clin Med.

[21] Hamada A., Soh J., Mitsudomi T. (2021). Salvage surgery after definitive chemoradiotherapy for patients with non–small cell lung cancer. Transl Lung Cancer Res.

[22] Ueno T., Yamashita M., Yamashita N. (2022). Safety of salvage lung resection after immunotherapy for unresectable non-small cell lung cancer. Gen Thorac Cardiovasc Surg.

[23] Li K., Cao X., Ai B. (2021). Salvage surgery following downstaging of advanced non-small cell lung cancer by targeted therapy. Thorac Cancer.

[24] Ceylan K., Kaya S., Samancilar O., Gursoy S., Ucvet A. (2011). The effects of neoadjuvant chemotherapy on pulmonary structures: a quantitative analysis. Thorac Cardiov Surg.

[25] Roberts J.R., Eustis C., Devore R., Carbone D., Choy H., Johnson D. (2001). Induction chemotherapy increases perioperative complications in patients undergoing resection for non–small cell lung cancer. Ann Thorac Surg.

[26] Doddoli C., Thomas P., Thirion X., Serée Y., Giudicelli R., Fuentes P. (2001). Postoperative complications in relation with induction therapy for lung cancer. Eur J Cardiothorac Surg.

[27] Li Z., He W., Wang C. (2022). ASO Author reflections: a simple method to predict risk factors of complications after pneumonectomy. Ann Surg Oncol.

